# Oral manifestations of ellis-van creveld syndrome. A rare case report

**DOI:** 10.4317/jced.55543

**Published:** 2019-03-01

**Authors:** Juan-Francisco Peña-Cardelles, David A. Domínguez-Medina, Jorge A. Cano-Durán, Daniel Ortega-Concepción, José-Luis Cebrián

**Affiliations:** 1 DDS, Oral Medicine Postgraduate in Complutense University of Madrid. Oral and Maxillofacial Surgery Service, Alcorcon Southern Hospital; 2 MD, Oral and Maxillofacial Surgery Service, Alcorcon Southern Hospital; 3 DDS, Oral Medicine Postgraduate in Complutense University of Madrid; 4 DDS, Oral Medicine Postgraduate in Complutense University of Madrid; 5 PhD, MD, DDS. Head of Oral and Maxillofacial Surgery Section of the La Paz University Hospital. Co-Director of the Service of Maxillofacial Surgery and Dentistry of Hospital la Luz. Head of the Oral and Maxillofacial Surgery Service, Alcorcon Southern Hospital

## Abstract

Ellis-van Creveld syndrome (EVC) or chondroectodermal dysplasia is an autosomal recessive disorder, characterized by dwarfism, polydactyly, hypoplastic fingernails and congenital heart defects, finding in most of the cases orofacial anomalies. We describe a clinical case of a 9 year old male patient diagnosed with EVC who visited our Maxillofacial private consultation at Alcorcon Southern Hospital, presenting typical oral manifestations such as dental agenesis, delayed eruption, hypoplasia of the enamel, dental dysmorphism, taurodontism and supernumerary teeth. EVC syndrome is a rare disease and requires a multidisciplinary approach. Oral features are constant and requires the jointly performance of Odontologist and Maxillofacial surgeon aiming to get an appropriate treatment sequence surgery-orthodontics in order to achieve a suitable functional result to improve the quality of life of these patients.

** Key words:**Ellis-Van creveld syndrome, chondroectodermal dysplasia, oral manifestations, craniofacial manifestations.

## Introduction

Ellis-Van Creveld syndrome or chondroectodermal dysplasia is a rare disorder, autosomal recessive (1-3), characterized by dwarfism, postaxial polydactyly of the hands and feet, severe dystrophy of the fingernails and congenital heart defects in about 50-60% of the cases (4-6).

First description of the syndrome was made by the Pediatricians Richard Ellis and Simon Van 433Creveld in 1940. It is the result of a genetic defect located in chromosome 4p16. (4) The prevalence varies from 1/1000000 in general population to 7/1500000 in amish population (Lancaster, Pennnsylvania, USA) (2,5).

Orally, the syndrome features consist of teeth of abnormal form (microdontia, conical teeth , dens in dente, taurodontism), supernumerary teeth, hypoplasia of the enamel, neonatal teeth in about 30% of the cases, as well as premature exfoliation of the teeth, malocclusion, multiple frenula, absent vestibular sulcus, submucous clefts, hypertrophic frena and labial frenula, dystrophic philtrum (6-8).

The present article describes the case report of a child patient diagnosed with EVC, presenting the major spectrum of oral features described in literature. Our patient was object of maxillofacial surgical treatment as a first stage sequence that will include combined orthodontic and restauration odontology. We emphasize the importance of a multidisciplinary approach for the correct management of these patients dental problems.

## Case Report

A 9 year old male patient was brought by his father, to our Maxillofacial private consultation at Alcorcon Southern Hospital, referred from Public medical institution, seeking for evaluation and possibility of treatment, the reason of consult was the abscense of eruption of the permanent superior incisors. The diagnosis of EVC was made at birth, in the public institution where he was referred from. There was no family background of the disease, he had healthy parents and brothers. The parents are originally from Morocco and consanguinity related in second degree.

Among the patient’s medical background, he presented heart congenital disease: interventricular communication that closed spontaneously and interauricular communication, surgically corrected at the age of three.

At the age of four, surgical correction of epispady was performed and at the the age of nine, surgical correction of both inferior limbs axis was performed “genu valgum deformity”.

At the clinical exploration, he presented short disarmonic stature (110 cm), both superior and inferior short limbs with bilateral hexadactyly and hypoplastic fingernails.

**Figure 1:**
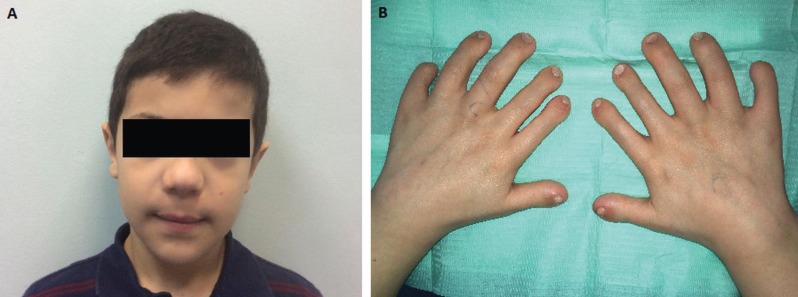
A. Extraoral vision, B. Intraoral vision: Polidactyly in both hands, as well as severe neil displasia.

Facial exploration, revealed normo-brachicephaly, normal eyelid fissures, wide nasal tip and narrow nostrils (Fig. 1).

**Figure 2:**
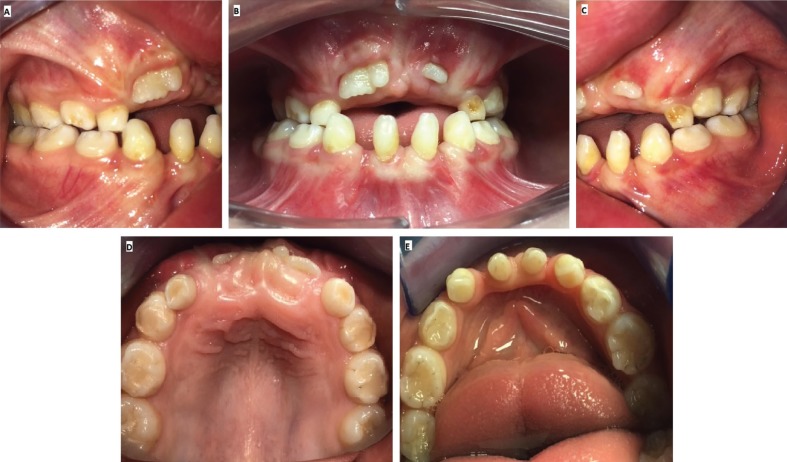
Intraoral vision A. Right side view, B. Front view, C. Left side view, D. Superior occlusal view, E. Inferior occlusal view.

Intraoral exploration revealed, agenesis of lateral superior and inferior incisors [12,22,32,42], dysmorfism compatible with conical teeth at inferior incisors and canines [31,33,41,43], fusion between a superior incisor (11) and a supernumerary tooth (mesiodens). As for the soft tissues, multiple frenum, macroglossia and glososquissis can be find (Fig. 2).

Furthermore, retention of maxillary incisors was present, due to the presence of a central supernumerary tooth. For this purpose, the retention was treated by extraction of the included central supernumerary tooth, under general anesthesia.

**Figure 3:**
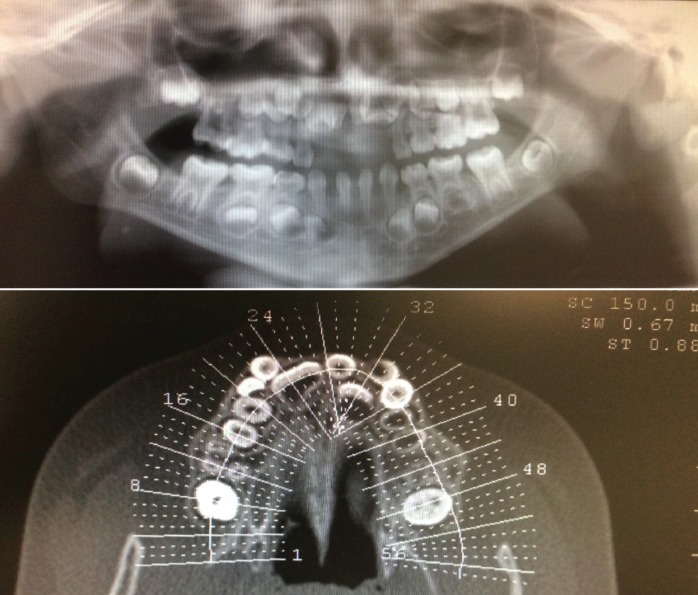
Orthopantomography and computer tomography. Both were performed with a diagnostic purpose prior to the surgery of mesiodens extraction.

Radiografically, certain degree of taurodontism was present at the permanent superior first molars and more discretely at the permanent inferior first molars (Fig. 3). In total 2 central supernumerary teeth were present. Maxillary compression was also evidenced

It is remarkable the maloclussion with a tendency to a class III and anterior crossbite.

## Discussion

In our reported case, there was no family medical pathologic background related, although there may be a history of consanguinity in up to 30% of cases.7

Among the published cases of the last decade, describing oral manifestations of the syndrome, the prevalence was 31.5% of men and 68, 42% of women ( Table 1).

**Table 1:**
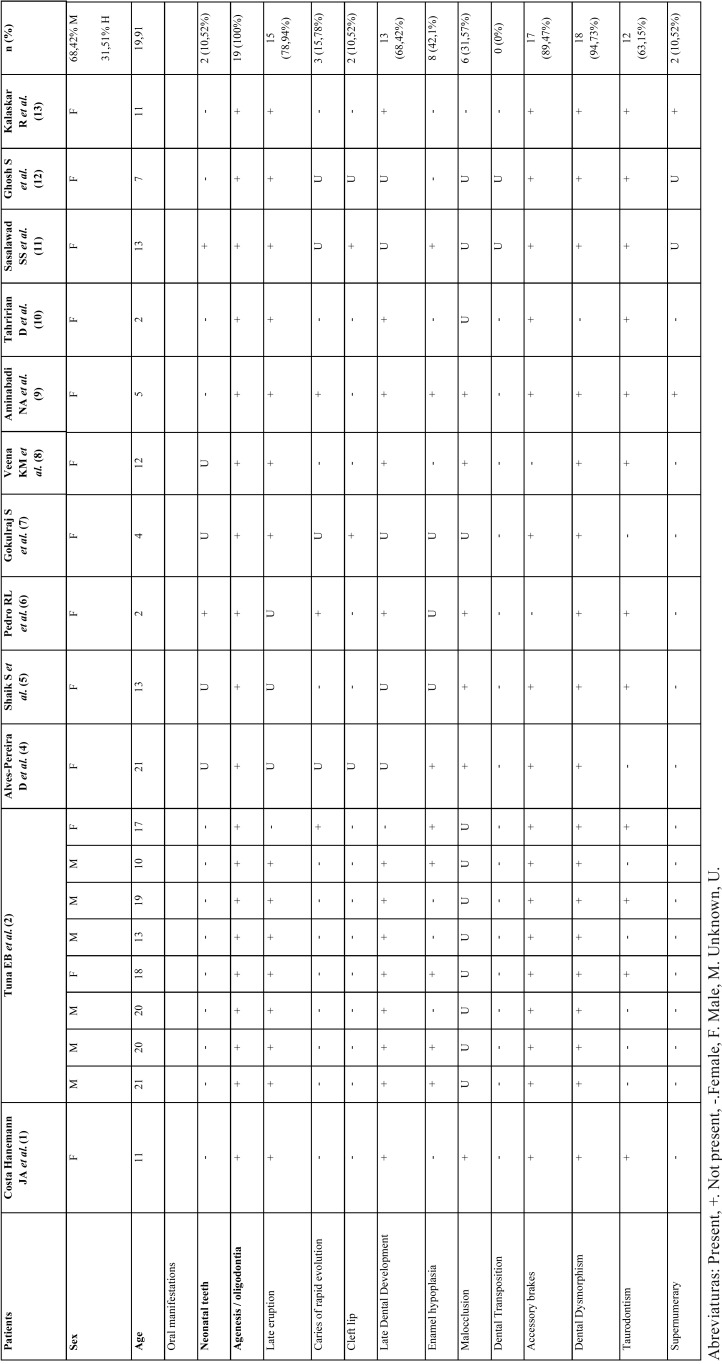
Clinical cases published in PubMed that collect oral manifestations in the last 10 years (4-13).

In the international literature, it is characteristic a tetrad, present in our patient, consisting of: dwarfism, bilateral polydactyly of the hands, ectodermal dysplasia (disorder in fingernails, teeth) and cardiac congenital malformations (2,4-6,10).

It is remarkable the varied spectrum of oral features involving both soft tissues and teeth, that are constant ( Table 1- Table 3), nevertheless there are unusual findings like the presence of taurodontism (Fig. 3) also present in our case. It is important to comment that development of taurodontism and conical form of teeth could be due to one single genetic alteration or multiple ones and be important to make differential diagnosis with other syndromes.

**Table 2:**
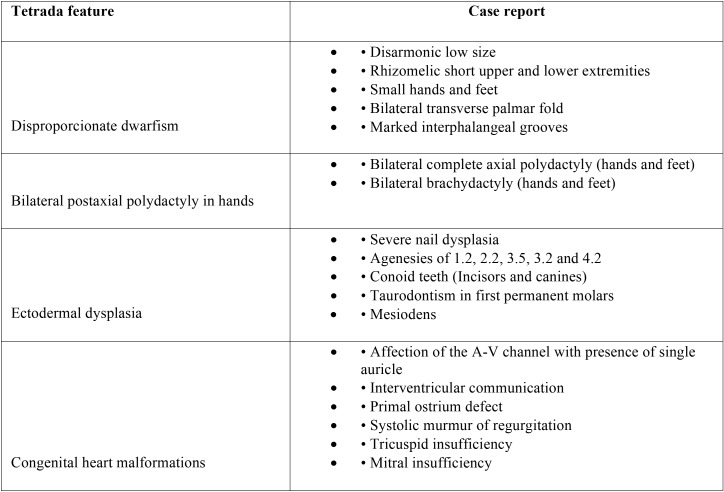
Tetrad characteristic in the case report.

**Table 3:**
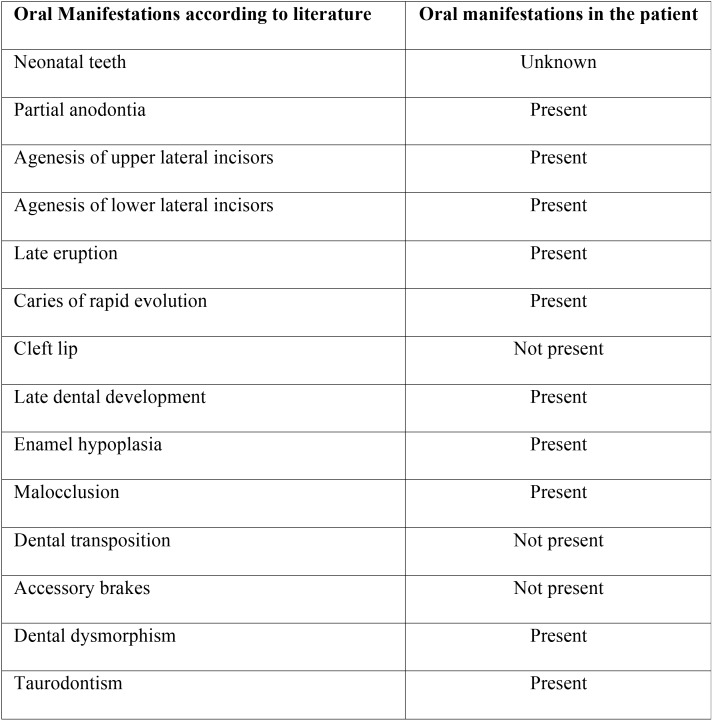
Oral manifestations of EVC syndrome in literature vs case report (1,5-8,12).

Another remarkable finding is the presence of maloclussion, specifically prognatism of the mandible 1; our patient presented a tendency to a maloclussion class III and anterior crossbite, due to maxillary compression and partial retention of permanent central incisors (Figs. 2,3).

In reference to the alterations of the dental eruption, an interesting fact present in this case is the poor root development in permanent first molars and in inferior canines, despite the patient’s age.

In general, the statistics shown in  Table 1, have coincided with literature, we also have another data reflecting results that help us to understand better the EVC. Case reports articles published in the last ten years describes oral manifestations evidence that dental agenesis has been seen in all cases (100%), the dental dimorphism in a total of 18 cases (94,73%), accessory frenums in 17 cases (89,47%) and late eruption in the 78,94% of cases, all of them present in the studied case. However, presence of supernumerary teeth is a characteristic which is in the present case but only in 2 of the 19 published cases (10, 52%).

## Conclusions

EVC syndrome is an infrequent entity and requires a multidisciplinary approach of specialists such as Dentist and Oral and Maxillofacial surgeon. It is important the maintenance of the dental health and to perform a correct dental diagnosis in order to establish an optimal treatment sequence.
